# A Family of ACO Routing Protocols for Mobile Ad Hoc Networks

**DOI:** 10.3390/s17051179

**Published:** 2017-05-22

**Authors:** Delfín Rupérez Cañas, Ana Lucila Sandoval Orozco, Luis Javier García Villalba, Tai-hoon Kim

**Affiliations:** 1 Group of Analysis, Security and Systems (GASS), Department of Software Engineering and Artificial Intelligence (DISIA), Faculty of Computer Science and Engineering, Office 431, Universidad Complutense de Madrid (UCM), Calle Profesor José García Santesmases, 9, Ciudad Universitaria, 28040 Madrid, Spain; delfinrc@fdi.ucm.es (D.R.C.); asandoval@fdi.ucm.es (A.L.S.O.); 2 Department of Convergence Security, Sungshin Women’s University, 249-1 Dongseon-dong 3-ga, Seoul 136-742, Korea; taihoonn@daum.net

**Keywords:** ant colony optimization, ACO, AntOR, bioinspired, mobile ad hoc networks, MANET, PAntOR, routing protocol, swarm intelligence

## Abstract

In this work, an ACO routing protocol for mobile ad hoc networks based on AntHocNet is specified. As its predecessor, this new protocol, called AntOR, is hybrid in the sense that it contains elements from both reactive and proactive routing. Specifically, it combines a reactive route setup process with a proactive route maintenance and improvement process. Key aspects of the AntOR protocol are the disjoint-link and disjoint-node routes, separation between the regular pheromone and the virtual pheromone in the diffusion process and the exploration of routes, taking into consideration the number of hops in the best routes. In this work, a family of ACO routing protocols based on AntOR is also specified. These protocols are based on protocol successive refinements. In this work, we also present a parallelized version of AntOR that we call PAntOR. Using programming multiprocessor architectures based on the shared memory protocol, PAntOR allows running tasks in parallel using threads. This parallelization is applicable in the route setup phase, route local repair process and link failure notification. In addition, a variant of PAntOR that consists of having more than one interface, which we call PAntOR-MI (PAntOR-Multiple Interface), is specified. This approach parallelizes the sending of broadcast messages by interface through threads.

## 1. Introduction

A mobile ad hoc network (MANET) [1] is a set of mobile nodes that communicate among themselves through wireless links. As opposed to conventional networks, a mobile ad hoc network does not need the existence of a previous infrastructure since each node relies on the others to communicate by creating the so-called multi-hop communication. This type of network has several drawbacks not found in conventional networks. For example, its topology can change quickly and unpredictably. Besides, variations in the capacities of nodes and connections may arise, as well as frequent errors in the transmission and a lack of security. Finally, the limited resources of nodes must be taken into account, since an ad hoc network will normally contain devices fed by batteries.

The MANETs are dynamically built when a set of nodes creates paths in order to obtain connectivity among them. The nodes in a MANET may not only act as a source or destination of a communication, but also as routers when a relationship between nodes cannot be achieved due to problems with reach. A routing protocol in an MANET requires a mechanism to be provided that maintains the routes towards the destinations given the movement of the nodes that may cause the destruction of the routes and that it is necessary to find an alternative route in order to keep the communication between the nodes. Routing protocols for MANETs are often called protocols of Level 2.5, since being generally found above linking protocols like IEEE 802.11 and below the network IP protocol. In MANETs, the conventional routing protocols will either have a very poor performance or will not be applicable. Alternative, new routing algorithms are therefore needed. In other words, mobile ad hoc networks need specific routing protocols because of their nature, as well as characteristics or requirements that they must meet to work properly, commenting also on the impossibility of using traditional solutions.

Ant colony optimization (ACO) is a branch of artificial intelligence (AI) that uses the concepts of swarm intelligence and takes its inspiration from the behavior of ants in nature (bioinspired). An essential aspect of the ACO routing is that the ants always show various full paths between the source and destination, increasing the overhead with respect to a purely reactive approach. A second characteristic is the way in which the ants choose the route. They construct the path hop by hop in a probabilistic manner using pheromone information. The use of this allows it to build on the acquired experience by the ants previously. This is the key to a highly distributed process. The fact that ants build their paths in a probabilistic manner allows the exploration of multiple routes. This makes the algorithm adaptable to changes in the network, increasing both the robustness (through the availability of reserve paths) and the throughput of the network. A third characteristic is the stochastic forwarding of the data packet based on the pheromone information, which ensures its routing by the best routes. If the pheromone keeps up-to-date by the use of enough ants, the load balancing follows changes in the network automatically. Due to its adaptability and robust properties, it also has become a paradigm for routing in mobile ad hoc networks. The ACO algorithms work iteratively. In every step, artificial ants build a solution in parallel with the problem in question, using the artificial pheromone matrix. Then, the pheromone matrix on the basis of the solutions found is updated. In this way, the pheromone matrix reflects information about good solutions that have been found to date, and it allows the ants of later generations to utilize this information to create new ones.

This paper consists of seven sections, with this Introduction being the first of them. The rest of the paper is structured as follows: Section 2 performs a review of the state of the art of the ACO routing protocols for mobile ad hoc networks observing that there are not representative protocols whose functioning metrics are degraded little or nothing in scalable environments. This review has included a comprehensive analysis of the AntHocNet protocol, the indisputable reference in the area. The study of the literature has also picked up a compilation of the major parallelization techniques of ACO algorithms, which is especially interesting if you want to provide a scalable solution. Section 3 comments on the AntOR-based protocol and its main phases. Section 4 presents a family of ACO routing protocols for mobile ad hoc networks: Disjoint Link Routes (AntOR-DLR), Restrictive Disjoint Link Routes (AntOR-RDLR), Unicast Disjoint Link Routes (AntOR-UDLR), AntOR-v2 and Hybrid ACO Routing (HACOR). Section 5 shows a comparative study between these protocols based on AntOR. Section 6 presents the main lines of research of this work. Finally, the conclusions are exposed in Section 7.

## 2. Related Work

We describe the most important ACO routing protocols, grouping these into proactive, reactive and hybrid.

The most representative proactive ACO routing protocols are the following:

Adaptive swarm-based distributed routing [2], better known as Adaptive-SDR, is inspired by AntNet. This protocol has the property of grouping the nodes in colonies to solve the problems of scalability of other protocols, derived from the fact that each node has to send an ant to others. The grouping of nodes in the colonies is done by a control central entity that becomes aware of their geographical positions. There are two types of ants: colony ants and local ants. The first ones have the mission to find routes from one colony to another. The local ants are internal to the colony, and they find routes within the colony, relying on two routing tables. This protocol has many drawbacks: many colonies are not advisable due to the overheads that occur; to know the optimal number of nodes that should be in a colony is not anything trivial; distributed systems do not always have a control central entity, as is presupposed; large processing of routing tables carried out by the local ants implies a high resources consumption, and it requires devices with important performances, and so on.

Mobile ant-based routing [3], better known as MABR, proposes a scheme to tackle the scalability problem of the routing in mobile ad hoc networks. This approach abstracts the network dynamic topology to get logical routers and logical links. These two concepts relate to the set of nodes and created paths among them, respectively. This algorithm uses the geographic partition of the area of the node and the geographical addressing of pheromone exploration. The problem with this proposal is only limited to presenting the theoretical model, not providing experimental results.

The probabilistic emergent routing algorithm for mobile ad hoc networks (PERA) [4] is a protocol that adjusts at each node the likelihood that each of its neighbors can receive and forward the data packet. The fact that forward ants are sent in broadcast mode from the source and intermediate nodes causes a multiple broadcast for finding different routes to the destination, causing a great overhead.

AntNet ring search and local retransmission (AntNet-RSLR) [5] is an adaptation from AntNet to mobile ad hoc networks through the incorporation of two techniques: Expanding Ring Search (ERS) and Local Retransmission (LR). Using the technique of the expanded ring searching, the request message of the route setup is spread progressively by flood from the source node. Initially, the message is spread to a small neighborhood with a small Time To Live (TTL) value, which is going to increase until you reach the destination. This message is forwarded by the source node if no response is received at a time interval. If the route requesting the TTL value has reached a certain threshold without receiving a response, it assumes that the destination is unreachable. However, this produces a great overhead and can cause loops that reduce the delivered packet ratio. To solve the problem of overhead, it introduces a variant of this technique called blocking-ERS, which does not assume the route search procedure from the source node when a new sending of the message in broadcast mode, generating a rebroadcast from an intermediate node chosen conveniently. A local retransmission technique is used when an intermediate node does not receive the corresponding data packet by expiating the timer value, sending a negative notification control message (Nack) for the intermediate node (and not the source node), returning to retransmit the failed data packet. This has the disadvantage that there is unknown a priori buffer capacity from the node that stores the data for its possible retransmission. Upon being based on a proactive protocol as is AntNet, the overhead should be present as a negative aspect, although the authors claim that it is reduced.

The most representative reactive ACO routing protocols are the following:

The ant-colony-based routing algorithm for mobile ad hoc networks (ARA) [6] is a reactive protocol in which the entries of routing table contain pheromone values that facilitate the choice of the neighbor. The authors affirm that, in the considered scenarios, the performance of this protocol is very similar to the DSR, presenting less overhead. However, it includes neither the scenarios representing a high network load, nor multimedia data.

DSR [7] is similar to Ad Hoc On-Demand Distance Vector (AODV) in that it forms a route on-demand when a transmitting node requests one. However, it uses source routing instead of relying on the routing table at each intermediate device.

The ant-based distributed routing algorithm for ad-hoc networks (ADRA) [8] is a reactive algorithm in which the ants move through the network between pairs of nodes chosen randomly. The authors assert that ADRA presents less average end-to-end delay, a lower overhead and a better delivered packet ratio than DSR. Furthermore, it allows them to optimize various QoS parameters, such as link quality, node load, and so on.

The improved ant colony optimization routing algorithm for mobile ad hoc networks (PACONET) [9] is a reactive routing protocol where the forward ants explore the paths in the network in search of paths from a source to a destination in restrictive broadcast mode, and the backward ants establish the acquired path by the forward ants.

The most representative hybrid ACO routing protocols are the following:

Mobile agent-based routing protocol for mobile ad hoc networks [10], better known as Ant-AODV, is a hybrid routing form based on ACO and the AODV routing protocol.

AntHocNet [11] is an adaptive, multipath and hybrid ACO routing algorithm. Data from 2004 and for almost ten years have had many extensions and variations to improve their performance. As mentioned above, AntHocNet is an algorithm hybrid (reactive and proactive), multipath and adaptive. AntHocNet follows a structure similar to AntNet Flying Ants (AntNet-FA), but it differs in its characteristics. The adaptive property, which occurs in AntHocNet, is suited to traffic and network conditions.

In the behavior of AntHocNet, the following phases are distinguished:

(i) Routing information setup: It starts with sending, on-demand, agents for the calculation of the path to the destination. This phase is supported by the property of multipath, which is considered of great importance, since they have to create the routes as soon as possible, so that the least number of data packets is lost.
(ii) Data routing: In this phase, data are sent in a unicast and stochastic manner using the pheromones of the routing tables, which have all of the nodes locally. This routing strategy aims to expand the data load, getting a better load balancing.
(iii) Path maintenance and the exploration of another new one: In this phase, while a data session is ready to relay the information, maintenance proactive ants are sent according to the data sending rate. The purpose of this phase is to upgrade the quality of the route links and the values of the pheromone between the path that goes from the origin to the destination.
(iv) Management of link failures: In this phase, the nodes can detect link failures. Once they are detected, AntHocNet tries to mitigate using different mechanisms like sending messages of failure notification and the route local repair.

The simulations analyze different scenarios in terms of the number of nodes, mobility and the density of nodes. In general, in all of them, AntHocNet has similar behavior to or even better than AODV. In particular, a better delivered packet ratio than AODV is obtained; the end-to-end delay is slightly higher in AntHocNet than in AODV for most simple scenarios (high density and short paths), improving in AntHocNet for more complex scenarios.

Ducatelle’s thesis [12] is an evolution of AntHocNet, regarding Di Caro’s thesis [11] and other works, such as [13]. The differences between both versions are in the use of different mechanisms in the route reactive setup process and the route maintenance proactive process.

HOPNET: The hybrid ant colony optimization routing algorithm for mobile ad hoc networks [14] is a hybrid routing algorithm based on ants hopping from one area to another. The algorithm has features extracted from the Zone Routing Protocol (ZRP) and DSR protocols, being highly scalable protocols compared with other hybrid protocols.

When a forward ant reaches a destination, a return ant (backward) is sent along the path discovered. The InterRT Interzone Routing Table (InterRT) zone stores the path to a node beyond its zone. This routing table is setup on demand, and the peripheral nodes are responsible for linking the zones. When the number of nodes is small, the continuous movement of the peripheral nodes does have to discover new routes constantly, causing more delay than in other hybrid routing protocols.

Table 1 shows a summary scheme of the analyzed protocols.

Others related works are [15,16,17].

In [15], the authors propose the biologically-inspired self-organized secure autonomous routing protocol (BIOSARP) showing its architecture, implementation and experienced results outdoors. These results show that BIOSARP is ant-based energy-efficient algorithm. However, the authors claim to be investigating other experiments of sensor networks in real environments, involving the mobility factor and security autonomous mechanism based on an artificial immune system (AIS).

In [16], the DDV-hop algorithm is proposed. This is an algorithm is a cluster algorithm that uses direction and velocity as the components of cluster formation. The results show that a good communication is established without system overheads.

In [17], the proposed adaptive routing protocol based on QoS and vehicular density (ARP-QD) is capable of finding a fast and reliable path for end-to-end data delivery within urban VANET environments according to diverse QoS requirements of different applications. Numerical simulations showed that ARP-QD has a higher delivery ratio than Greedy Perimeter Stateless Routing (GPSR) and Receive on Most Stable Group-Path (ROMSGP), without making a large compromise on the delivery delay; however, it has the disadvantage that they do not take the real data trace into consideration to validate the ARP-QD protocol and combine the link correlations to estimate link quality.

## 3. AntOR-Based Protocol

AntOR is inspired in the AntHocNet algorithm, more specifically, in the specified version by the Ducatelle’s thesis [12], inheriting its characteristics of hybrid (reactive and proactive), multipath and adaptive protocol. Like its predecessor, it presents the following phases:

- Routing setup: When starting the data session, the source node, on demand, sends agents to discover the available routes to the destination.
- Data routing: the data are sent out through the nodes to the destination using the route information, being able to utilize the multi-hop technique, i.e., sending data through intermediate nodes that act as routers.
- Established path maintenance and exploration of new routes: it is the updated information of existing routes, and it tries to discover new ones. This phase consists of two stages: pheromone diffusion and ant proactive sending.
- Management of link failures: These occur because a node is out of the reach of the network or because it does not receive control messages, which are responsible for informing a node of its closest neighbors.

In so far as the main differences of AntOR regarding AntHocNet, basically these consist of the introduction of the following elements/processes:

- Specification of the disjoint link or node routes.
- Separation between the pheromones in the diffusion process.
- Using the distance metric in path exploration.

These three characteristics influence especially Phases 1 and 3 of the algorithm, that is in the setup phase and maintenance and exploration of new routes.

### 3.1. Data Structures

Like almost all of the ACO routing protocols, AntOR requires two data structures: the routing table and the neighbor table. These have a similar functionality as other routing protocols. Each of them is specified.

#### 3.1.1. Routing Table

Like all routing ACO algorithms for mobile ad hoc networks, the information related to routing is organized into the so-called routing tables. This data structure is present also in ACO routing protocols for wired networks as AntNet or routing classic protocols for mobile ad hoc networks as AODV.

These tables contain the utilized information by the algorithm in its forwarding of local decisions. The kind of information contained, as well as the way in which it is used and updated depend solely on the characteristics of the algorithm. The routing table is in turn a local database and a local model of the global state of the network.

This table consists of the following fields:

- Regular pheromone ($\tau_{\mathrm{ij}}^{d}$): It indicates the path through which the data travel. It is a heuristic value that contains an estimate of goodness to relay data packets along the route that goes from *i* to the destination *d* with next hop *j*. This value is expressed as the inverse of a time estimate or cost, as will be explained when Equation (7) is introduced. This cost is based on the metric used for the algorithm evaluation.
- Virtual pheromone ($\omega_{\mathrm{ij}}^{d}$): It indicates a path that can possibly be good. This virtual heuristic value has the mission of the auxiliary value and is utilized as an alternative. It is created or updated in the pheromone diffusion process.
- Average hop number ($h_{\mathrm{ij}}^{d}$): It is utilized in the local route repair process to indicate correctly how long the process needs to run.

The regular pheromone and average number of hops values are related in the following way: when a route has a value from one, it also has that from the other. This is due to the fact that these two values are involved in the use of the backward ants in the reactive process, the proactive (exploration of new alternative routes) and the local route repair. However, the virtual pheromone value is created or updated independently, because it is utilized in the pheromone diffusion process.

Table 2 shows the structure of the routing table in AntOR. This structure stores the following information for each entry: reachable destination of the data session, next hop to which the data are routed, the value of the regular pheromone and virtual pheromone and the average number of hops.

This table grows dynamically according to the reachable routes that are calculated.

#### 3.1.2. Neighbor Table

This data structure contains the information that each node has of the one-hop neighbors with its corresponding listening time. The neighbors’ table maintained by the node *i* is a vector with an entry for each one of its neighbors. Each entry corresponds to the information that the node *i* has in the presence of the neighbor node *j*, as well as a time value that indicates when it was the last heard from, that is that *i* received a message from *j*. This structure is utilized, as its name suggests, to indicate the presence of the neighbors and to detect possible link failures.

Table 3 represents the generic table from the neighbors of AntOR. In this structure, every local node has a list of one-hop neighbors with the following information: a neighbor identifier Id $Neigh_{k}$ and the last time value Time $Neigh_{k}$, associated with the notification message from vicinity (Hello), which the neighboring node sent.

### 3.2. AntOR Phases

#### 3.2.1. Route Setup

Initially, the nodes do not have routing information to send the data, but they have applications to start: traffic generator, ftp, ping, ⋯, the network interfaces, the protocol stack (IP, UDP/TCP, and so on). The application generates data in the node, but having no available route, cannot send them. The node needs, therefore, to send reactive agents (reactive ants) to discover the routes to the destination.

##### Reactive Forward Process

At the beginning of the route setup process, the node *s*, the source of the session data, creates an Reactive Forward Ant (RFA). This ant is a control packet, which aims to find a path from *s* to a given destination *d*. This ant goes from the source node to the destination node, being sent by *s* in broadcast mode.

The intermediate nodes that receive this ant forward it in the route searching process until reaching the destination. This type of ant has a list *P* of visited nodes so that intermediate nodes are not repeated.

The forwarding mode of the RFA at the intermediate nodes may be unicast or broadcast, depending on if the current node has available routing information from the destination *d*. In general, the RFA is sent in broadcast mode, because it aims to discover the first route. Unicast mode is utilized whenever the current node has information of a neighboring router that serves to relay the correspondent RFA to the next hop. In other words, a node has routing information whenever the route setup is done, utilizing the first setup in broadcast mode for the sending of RFAs and in the subsequent ones (because of link failures at source nodes), this mode or unicast, due to the remaining routes belonging to other previous setups.

Unicast forwarding is performed utilizing Equation (1) probabilistically, where $\tau_{in}^{d}$ is the regular pheromone value of the link that goes from node *i* to the next hop *n* in route to the destination *d*; $N_{i}^{d}$ is the set of neighbors of node *i* with a available route to *d*; and $\beta_{1}$ is a setting parameter influencing the exploratory behavior of ants.

(1) $$P_{in}^{d}=\frac{{\left(\tau_{in}^{d}\right)}^{\beta_{1}}}{\sum_{j\in N_{i}^{d}}{\left(\tau_{ij}^{d}\right)}^{\beta_{1}}}\;\beta_{1}\geq 1$$

The value $\beta_{1}$ is determined experimentally. If we utilize a high value of $\beta_{1}$, the routes with a higher pheromone regular concentration are the candidates to relay the RFAs, obtaining the initial route quickly. If, on the contrary, we set to a lower value, routes tend to be chosen with similar probability.

More in detail, the route selecting process of Equation (1) is as follows:

When a node has the possibility of doing the hop to its neighboring nodes to get the destination *d*, it calculates the probabilities $P_{in}^{d}$ of each of these neighbors *n* with the regular pheromone value. According to this strategy, we do not choose the routes a priori that we are going to utilize, but we select them as follows:

- It is generated with a random number rand with uniform probability between zero and one.
- The non-overlapping associated intervals with the calculated probabilities $P_{in}^{d}$ are calculated above. These intervals are associated with each possible neighboring node at the time of selecting the candidate to transmit the message.
- Once obtained rand, the associated route is chosen with the interval that corresponds to $P_{in}^{d}$. For this, the reactive ants are forwarded to the next hop *n* having as destination *d*.

Equation (1) is based on a selection mechanism, widely utilized in genetic algorithms, called the roulette selection. This mechanism is also known as the fitness proportionate selection; where *N* is the number of existing individuals and $f_{i}$ the fitness of the *i*-th individual, the associated probability of its selection is given by the following equation:

(2) $$p_{i}=\frac{f_{i}}{\sum_{j=1}^{N}f_{i}}$$

##### Reactive Backward Process

Upon reaching the destination, the RFA becomes an Reactive Backward Ant (RBA). The latter follows the list of visited nodes generated by RFA to return to the source node *s*. In this process, only the first copy of the forward ant coming is chosen, discarding the remaining. In this way, a unique route is set, and as mentioned previously, the overhead is reduced.

Artificial ants are inspired by natural ants, but have some additional capabilities that improve their performance. Therefore, while natural ants deposit pheromone as they are going as they return, the artificial ants have an internal memory where tour nodes’ information is stored. This information is utilized by the backward ants in the return, the reason why the return of the ant to the source is done in unicast mode. In this trip, the backward ant is responsible for creating or updating a record in the routing table. This registry stores a value that represents the inversion of the cost in terms of estimated time to send a data packet from the destination node to the source through intermediate nodes.

Incrementally, the backward ant calculates an estimate or cost $c_{i}^{d}$ of time that it would take a data packet to travel through that list *P* of nodes to the destination *d* from the node *i*, updating the routing tables.

The updating process from the routing table registry is as follows: the backward ant updates the number of hops $h_{in}^{d}$ and the regular pheromone value $\tau_{in}^{d}$ from the routing table registry, being *n* the previous visited node, *i* the current node (which is currently being processed) and *d* the destination of the session.

Equation (3) summarizes the updating process of the number of hops $h_{in}^{d}$:

(3) $$h_{in}^{d}\leftarrow \alpha h_{in}^{d}+\left(1-\alpha \right)h\;\alpha \in \left[0,1\right]$$

In this equation, *h* is the number of hops that the backward ant has traveled and α a regulation parameter that indicates how fast the formula to the new information is adapted. In experiments, α has been always set to the usual value of 0.7.

The regular pheromone update process is as follows:

The estimate $c_{i}^{d}$ commented on previously is calculated according to Equation (4), that is it comes to be the sum of the time estimates that it takes to reach the next hop at each node of the list *P*:

(4) $$c_{i}^{d}=\sum_{i=1}^{n-1}{\hat{T}}_{i\to i+1}$$

The value of the local estimate ${\hat{T}}_{i\to i+1}$ is defined as the product of two terms:

- The current number of packets in the queue, which are ready to sent at the MAC layer plus one, that is:
  $$Q_{mac}^{i}+1$$
- The required average time to send a packet:
  $${\hat{T}}_{mac}^{i}$$
  with what ${\hat{T}}_{i\to i+1}$ is, as shown in the Equation (5):
  (5) $${\hat{T}}_{i\to i+1}=\left(Q_{mac}^{i}+1\right){\hat{T}}_{mac}^{i}$$

If we consider the real-time $t_{mac}^{i}$ that it takes a node to send a packet:

(6) $${\hat{T}}_{mac}^{i}\leftarrow \eta {\hat{T}}_{mac}^{i}+\left(1-\eta \right)t_{mac}^{i}\;\eta \in \left[0,1\right]$$

In the experiments, η has also been set to 0.7. This value is determined experimentally observing the best behavior of the algorithm. With this parameter, we want to indicate that ${\hat{T}}_{mac}^{i}$ has more priority than $t_{mac}^{i}$, specifically 70%. The value $t_{mac}^{i}$ at each hop is estimated in AntOR as the time difference between the sending and receiving of the backward ant.

Finally, the updating of the regular pheromone value is calculated as shown in Equation (7):

(7) $$\tau_{ij}^{d}\leftarrow \gamma \tau_{ij}^{d}+\left(1-\gamma \right){\left(c_{i}^{d}\right)}^{-1}\;\gamma \in \left[0,1\right]$$

Using the previous equation, the value of a registry $\tau_{ij}^{d}$ of the routing table from node *i* is updated, being *j* the next hop, *d* the destination that we want to reach and γ a setting parameter set to 0.7 in the performed experiments. This is determined experimentally observing the best behavior of the algorithm.

In the particular case, there is virtual pheromone in the link/arc that we want to update (as a consequence of which the diffusion process is completed before the route setup process), that is if the node *i* that has a route to the destination *d* using next hop *j* already has the virtual pheromone, the update of the regular pheromone in the route setup process described by Equation (7) is, for the process of regular and virtual pheromone separation, as follows:

(8) $$\begin{array}{c} Regular_{final}=F\left(Regular_{new},time\right)\\ Virtual_{final}=0 \end{array}$$

being:

$$Regular_{final}=F\left(Regular_{new},time\right)$$

a simplified representation from Equation (7).

In other words, when we get a new regular pheromone value, the pheromone virtual value is set to zero. We give priority, therefore, to the regular pheromone regulated against the virtual one, since data are only routed along routes with regular pheromone values. Thus, we do not originate any conflict in the creation and maintenance (updating) of the routes, the algorithm being optimized with regard to its capacity (internal memory) due to the route table only having an entry of the destination and next hop. This entry can contain its corresponding field of pheromone, regular or virtual, but not both.

#### 3.2.2. Data Stochastic Routing

The first route setup creates a unique path between the source and destination, as shown in the routing table. Other route discoveries and the route exploration, which is explained in the following section, originate multiple paths between the source and destination. This is carried out such that the data can be forwarded in mode multi-hop according to a probabilistic technique based on the routing tables. The strategy consists of making the data load expand through load balancing. This is important in mobile ad hoc networks because the wireless channel bandwidth is very limited.

The data routing is given by the following equation:

(9) $$P_{in}^{d}=\frac{{\left(\tau_{in}^{d}\right)}^{\beta_{2}}}{\sum_{j\in N_{i}^{d}}{\left(\tau_{ij}^{d}\right)}^{\beta_{2}}}\;\beta_{2}\geq 1$$

Equation (9) is similar to (1). The difference is in the exponential parameters $\beta_{1}$ and $\beta_{2}$.

#### 3.2.3. Established Path Maintenance and Exploration of New Routes

As its name suggests, this phase consists of a proactive process of established route maintenance and exploration of new routes, which updates and expands the available routing information. This allows us to build multiple routes that serve as support for the created initial route in the reactive route setup process. This proactive process contemplates two subprocesses: pheromone diffusion and proactive ant sending.

This phase of AntOR differs from the similar AntHocNet in the separation of pheromones in the diffusion process, the disjoint ability and the use of the distance metric, differences that directly affect the two subprocesses of this phase.

##### Pheromone Diffusion

Pheromone diffusion aims to expand the information available from the pheromone in the network by sending updating periodic messages and the bootstrapping technique to know reachable destinations in the network. This process is similar to pheromone diffusion in nature. The Hello messages play an important role: every certain interval of time *t*, the nodes send messages of this type in broadcast mode. The experiments *t* was set equal to one second. These messages are also used to know the one-hop neighbors and to detect link failures. At the same time, these messages serve to spread the necessary pheromone in the bootstrapping process.

This generated Hello message is as follows:

A node *i* chooses a maximum number *k* of destinations by consulting the information in its routing table. When there are more available destinations, these are selected randomly. For each destination *d*, the Hello message has information of the best pheromone value $v_{i}^{d}$ that the node *i* has for the destination *d*. This is calculated by taking into account all of the possible regular pheromone values $\tau_{ij}^{d}$ and virtual pheromone values $\omega_{ij}^{d}$ associated with the destination *d*. In addition to including it, it is indicated with a flag if the chosen value corresponds to a regular or virtual pheromone value.

Once created, the Hello message is sent, as described earlier, in broadcast mode. All of the neighbors of the node that sends this message Hello receive a copy. Thus, a neighboring node *j*, upon receiving this message, estimated a new value that indicates how good the route from this node *j* to the sender *i* is, which has a reachable destination *d*, shown in the destination list of the Hello message. This estimation is made by combining (bootstrapping) the pheromone value $v_{i}^{d}$ from the Hello message with the local estimation or cost $c_{j}^{i}$ of the hop *j* to *i*, i.e., the link between the node *j* and the node *i*.

Equation (10) summarizes the bootstrapping process:

(10) $$k_{ji}^{d}={\left({\left(v_{i}^{d}\right)}^{-1}+c_{j}^{i}\right)}^{-1}$$

being $k_{ji}^{d}$ the bootstrapped value obtained in this process. Thanks to the use of this technique, the overhead is low, because the needed uniqueness is to send the value $v_{i}^{d}$ from node *i* to *j*. This information is included in the Hello message, which is sent in broadcast, and when it is received by a node, it never is forwarded (it would increase overload then).

This bootstrapping process is repeated constantly when the simulation starts with the sending of Hello messages in an asynchronous way by each node in the network. Although this process has low overhead, it may have reliability problems. The value obtained by bootstrapping only is correct when the value $v_{i}^{d}$ is contained in the Hello message. This is especially problematic in highly dynamic environments where routing information is not updated quickly and, especially, if the included value in the Hello message corresponds to the virtual pheromone. To this problematic situation we should add the fact that the bootstrapping process is relatively slow, because the sending of Hello messages is carried out every certain interval time (in order to maintain its efficiency).

For the above considerations, AntOR has the premise that the bootstrapped pheromone value $k_{ji}^{d}$ obtained in Equation (10) is not very reliable. This feature affects directly the update of the routing table when we use this value $k_{ji}^{d}$ and the separation of regular and virtual pheromone values. Generally, the virtual pheromone value is updated with the new bootstrapped pheromone value. On the other hand, the regular pheromone is only updated by this bootstrapped pheromone value ($k_{ji}^{d}$) when the following conditions occur simultaneously:

(a) The node *j*, which receives the corresponding Hello message, has a non-zero regular pheromone value.
(b) This Hello message also contains a value $v_{i}^{d}$ corresponding to the regular pheromone.

In addition, AntOR applies the following premise:

If node *j*, which has a route to the destination *d*, already has the regular pheromone and it gets the virtual pheromone contained in the Hello message during the pheromone diffusion process, then the virtual value is not updated at node *j*, since it cannot simultaneously have non-zero values in both pheromones. The value of the final virtual pheromone is, therefore, zero.

In addition, it should be noted that the ants from pheromone diffusion also serve to detect broken links.

##### Ant Proactive Sending

Exploration consists of a process to discover new routes that serve as alternatives for the sending of data packets. The diffusion process discussed earlier is essential for the correct functioning of the exploration. Initially, it generates the corresponding proactive forward ant PFA for subsequent sending. These ants are never sent in broadcast mode, since they only go by paths that have marked the route, either by the regular pheromone or by the virtual pheromone.

In AntHocNet [12], the equation of route exploration is as follows:

(11) $$P_{in}^{d}=\frac{{\left[max\left(\tau_{in}^{d},\omega_{in}^{d}\right)\right]}^{\beta_{3}}}{\sum_{j\in N_{i}^{d}}{\left[max\left(\tau_{ij}^{d},\omega_{ij}^{d}\right)\right]}^{\beta_{3}}}\;\beta_{3}\geq 1$$

In AntOR, the equation of exploration is as follows:

(12) $$P_{in}^{d}=\frac{{\left(\psi_{in}^{d}\right)}^{\beta_{3}}}{\sum_{j\in N_{i}^{d}}{\left(\psi_{ij}^{d}\right)}^{\beta_{3}}}\;\psi \in \left\{\begin{array}{c} \omega \;virtual\\ \tau \;regular \end{array}\right.$$

where ψ is a regular or virtual pheromone value and $\beta_{3}$ a setting parameter relative to the influence of the pheromone concentration (with similar functionality to $\beta_{1}$ and $\beta_{2}$).

It is worth mentioning that AntOR uses the distance metric, a circumstance that does not occur in AntHocNet [12]. Thus, the number of hops from the best routes found is considered. In this way, a proactive ant is controlled and cannot go to more nodes than those set by the so-called hop limit, which is set according to the best routes (those with less distance in number of hops) calculated above. The reason for choosing this metric (and not others, such as the delay, for example) is that it is considered stable, since it does not influence the interference caused by other devices.

#### 3.2.4. Management of Link Failures

Nodes can detect link failure in a unicast transmission or when a Hello message is expected and is not received. When a link fails, the node can lose the route to one or more destinations. An example of a link failure occurs when a neighbor moves beyond the transmission range. Link failures consider two kinds of problems:

- If the node has other alternatives to the destination or if the route to the destination is lost, because it has not been used regularly, it has to be notified with a link failure message.
- If the route to a destination that is regularly used by the data is lost and is the only alternative of the node, the loss is especially important, and the node tries to repair the path locally.

As its name suggests, AntOR-DLR [18] is derived from the basis protocol AntOR with the only restriction that its specification takes into account only routes that do not share links.

Table 4 shows the routing table of AntOR-DLR. As we can see, the routing table adds, regarding AntOR, an additional field called disjoint session. The basic idea to find and to represent disjoint link routes consists of marking each disjoint link with a label indicating what the source of the data session is. This mark is indicated in the field disjoint session in the routing table discussed earlier.

**Figure 1:**
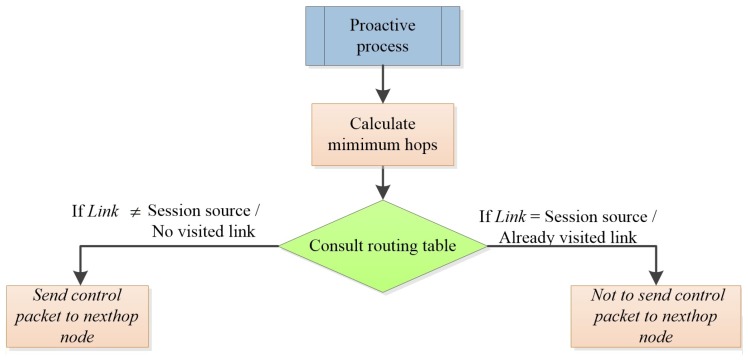
Flowchart of disjoint link routes (AntOR-DLR).

Figure 1 shows the flowchart of the procedure of the calculation of disjoint link routes. As we can observe, the procedure is as follows: the disjoint session field in the routing table is consulted to verify if the link is already disjoint or not. To this end, we check if the link is associated with the source of the data session. In the negative case, it sends the corresponding proactive forward ant to the previously calculated next hop. Upon receiving this proactive ant, the process is repeated in the intermediate nodes.

As its name suggests, AntOR Disjoint Node Route (AntOR-DNR) [18] is derived from the basis protocol AntOR with the only restriction that in its specification, it takes into account only routes that do not share nodes.

Like AntOR-DLR, the routing table of AntOR-DNR adds an additional field, the so-called disjoint session with respect to AntOR.

The main difference between AntOR-DNR and AntOR-DLR consists of the way of calculating the routes in the exploratory process: in the disjoint node routes, it is the node responsible for detecting the disjoint property, while for disjoint link routes, is the own link.

**Figure 2:**
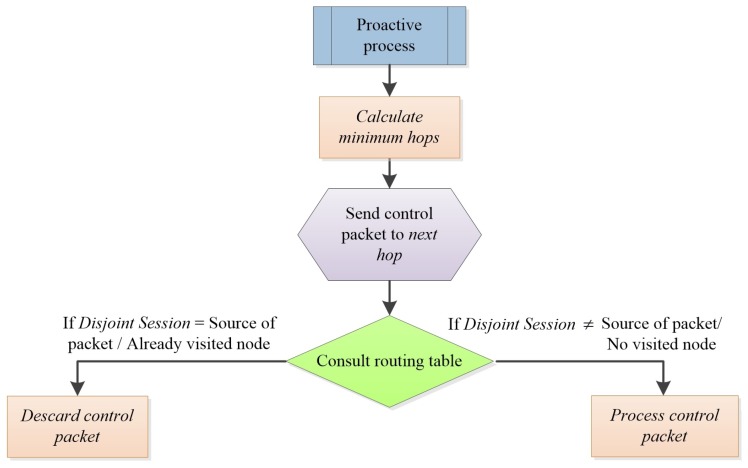
Flowchart of disjoint node routes (AntOR-DNR).

Figure 2 contains the flowchart of the functioning of AntOR-DNR. The protocol works as follows. Initially, the corresponding proactive forward ant is sent to the next hop by applying Equation (12). When the node receives the ant, it consults its routing table to see whether the field disjoint session has the same value as the source of the ant. In the case of having the same value, we discard the packet, because it is treated as a node disjoint route.

## 4. AntOR Protocol Family

As its name suggests, AntOR-RDLR [19] is derived from the protocol AntOR-DLR, presenting two important differences with respect to this. The first, which gives rise to its name, is that in the route established maintenance phase and exploration of new routes occurs, where, on the one hand, enabling proactive ants and the coexistence of non-disjoint link routes to adapt flexibly and disjoint link routes and, on the other, restricting these to contain a maximum number of disjoint links. The second difference occurs in the route setup phase and is related to the pheromone update process.

The pheromone update process in the route setup phase is as follows: If the node ithat has a route to the destination d already has a value of the virtual pheromone and in the route setup phase, we obtain the other regular pheromone applying Equation (7), then the value of the regular pheromone replaces the virtual pheromone using the maximum of these two values and setting the value of virtual pheromone to zero. Equation (13) summarizes the process:

(13) $$\begin{array}{c} Regular_{last}=F\left(Regular_{new},time\right)\\ Regular_{final}=max\left(Regular_{last},Virtual_{old}\right)\\ Virtual_{final}=0 \end{array}$$

As its name suggests, AntOR-UDLR [20] is derived from the protocol AntOR-DLR, differing from this in the link failure management phase.

AntOR-UDLR replaces notification messages sent in broadcast mode in AntOR-DLR by simple messages (unicast) sent to the predecessor, which has a valid route to a reachable destination, understanding a valid route to belong to the active session of a given destination with a positive value of the regular pheromone.

**Figure 3:**
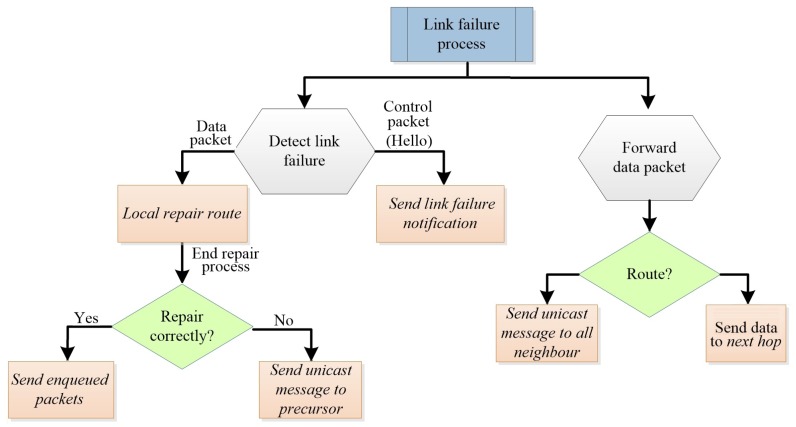
Link failure management (AntOR-UDLR).

Figure 3 illustrates the notification process of the failure of the link in AntOR-UDLR.

When there is a node failure, both link and node failures occur. The node that perceives the failure eliminates this from its neighbor table to the corresponding node. Then, it updates the routing table with the new information of the pheromone and proceeds as follows:

(a) If there is no route at the source, a reactive forward ant is sent.
(b) If there is no route at the intermediate node and a data packet was retransmitting when the failure occurred, it sends a route repair forward ant. If there is no reply from the corresponding repair backward ant in a determined period of time, a unicast message is sent to the precursor of the route, informing that the destination is unreachable. The node that receives this message updates the routing table and forwards this message to the predecessor, and so on, up to the source node of the data session.
(c) If there is no route at the intermediate node and if the control packet (a Hello message or a unicast control message) is dealt with, no message is sent. This can cause routes to not be repaired properly. When an intermediate node, which routes the data, does not find a valid route, it sends a unicast message to all one-hop neighbors to update their routing tables. It is necessary to send this message to all neighbors because, otherwise, in addition to the fact that there is not a located valid route, there is also no information of the predecessor. When one of these neighboring nodes has a valid route to the destination, it forwards the unicast message to the precursor of the route, and so on, until reaching the source node.

As its name implies, AntOR-v2 [21] is derived from the protocol AntOR (more specifically of AntOR-DLR), although there are important differences, namely: control packet buffering, obsolete route management, management of sending failures and removal of the virtual pheromone in the maintenance of established routes and the exploration of new routes phase. these differences are discussed in detail.

The control packet buffering consisting of these is stored for subsequent sending to the respective destinations at every certain interval of time. This fact allows it to have synchronism in the sending of packets and not to congest the network, decreasing its collision. Each entry in the buffer includes the following information: (a) the socket that sends the packet; (b) the control packet or particular message of the protocol; and (c) the destination address (it can be a broadcast address or unicast address sent to a specific node).

The obsolete route management replaces the evaporation process of the pheromone. This event takes place every certain interval of time and is as follows:

- Each entry in the routing table has a field (timestamp) indicating when it was created or was last updated.
- If the field timestamp associated with each route in the routing table is lesser than the difference between the current time and a given time limit, the aforementioned entry is removed in a local way (each node).
- The value of this limit is important. A low value makes the routes converge slowly, eliminating routes to active destinations. Conversely, a high value implies a high convergence in the creation of routes with the consequent possibility of maintaining obsolete routes.

The management of sending failures is related to a fault tolerance. When a failure is detected in a control message, a neutralization process is launched. In highly dynamic environments (with more links breaks), the number of neutralization processes as a route local repair is greater, causing a major overload. The introduced mechanism pretends to alleviate this fact by checking for the existence of a valid path (positive value of regular pheromone) to the neighbor that will be transmitted. Only in the case that the path exists, the control packet is sent.

The fourth and final difference and, perhaps the most significant, is, as mentioned above, the elimination of the virtual pheromone in the maintenance of established routes and exploration of new routes phase. It is intended to reduce the overhead using proactive agents that do not require routes with the virtual pheromone. These agents create alternative routes that go from neighbor to neighbor until reaching the destination node. At the time of selecting the next hop, the agents take into account the maximum value of the regular pheromone from the one-hop neighbor. In this way, we can reach alternative routes, which are also link disjoint.

HACOR [22] consists of a refinement of AntOR-v2, differing from this in the incorporation of the data packet buffering capacity, in an optimized link failure neutralization process and the introduction of a particular type of S-ACO during the maintenance of established routes and the exploration of new routes. These differences are discussed in detail.

The data packet buffering has these stored for subsequent sending to their respective destinations at every certain interval of time on the assumption that there are no routes. Indeed, when the data packet is ready to send to the next hop, it checks if there is a valid route to the destination belonging to the current data session. In the case that there is not a valid route, the data packet is stored in the packet queue, sending a local route repair forward ant to solve the problem. At the same time, repairing the route is tried; the node sends a unicast message to all reachable neighbors. The neighbors, which receive this message, send it to its predecessors. Otherwise, that is, if there is a valid route, it is processed for sending.

The first event that occurs when there is a node failure is that the node that perceives it updates its neighbor table, eliminating all routes that have the node that fails as a next hop. If there is not a route at the source node, the route setup is started, sending a reactive forward ant. If there is not a route at an intermediate node and a data packet was being forwarded when the failure occurred, a local route repair forward ant is sent to each of the destinations of all affected data sessions. If there is no route at the intermediate node and a control packet (Hello) was being sent in broadcast mode, no neutralization process is performed. If a unicast control packet was being forwarded, a Unicast Link Notification (ULN) message is sent to the predecessor node. This process is repeated until we reach the source node.

Finally, the third distinctive feature of HACOR with respect to its predecessor is the introduction of a variant of Simple Ant Colony Optimization (S-ACO) in the maintenance of established routes and the exploration of new routes phase, which basically consists of the following:

(a) The virtual pheromone leaves, as is necessary at this phase.
(b) We do not utilize the evaporation process.
(c) A free-loop method is used when the proactive forward ant (PFA) has come to the destination node. Subsequently the loop is removed, this PFA becoming a free-loop PBA, which returns to the source by the visited nodes in the list, updating the routing tables of each node.
(d) We do not need the initial establishment of pheromone values to each one-hop neighbor. The exploration process is done hop-by-hop with the pheromone information that has the one-hop neighbors using the Hello messages. Each node that receives a Hello message from another one-hop neighbor updates its route with the new value of the pheromone.
(e) The proactive forward ants utilize the link-disjoint route.
(f) This use of the disjoint routes involves the checking of whether the one-hop neighbor that has to forward the corresponding proactive agent belongs to a disjoint route or not. In the case that the neighbor belongs to a disjoint route, it is not chosen (in order to reduce the overhead).

As its name suggests, PAntOR [23] derives from AntOR, more specifically it can be considered a parallel approximation of AntOR-DNR. The reason for choosing AntOR-DNR (and not AntOR-DLR) is that it is intended to analyze the worst case, hence the choice of the first for being more restrictive.

Previously to PAntOR’s specification, it is advisable to point out some aspects of the parallelization of the ACO algorithms.

Firstly, it should be known that practically all parallel work on ACO algorithms is designed for centralized systems based on the technique of master-slave, where the central master distributes work to other processors. PAntOR, on the other hand, is designed for decentralized systems, which gives it even greater relevance.

Secondly, it is good to know that the parallel ACO algorithms are classified according to the two criteria described below:

A possible classification difference exists if a parallelization of an ACO algorithm is standard or is especially designed. A standard ACO parallelization aims to decrease the run time without changing the behavior of the algorithm. On the contrary, specific parallel algorithms change ACO in order to obtain a more efficient algorithm. A method utilized to differentiate between these two approaches consists of how it makes use of the exchange of information between processors.

Another possible classification checks if the algorithm has a centralized or decentralized approach. In a centralized approach, it is normal that a processor collects information of the pheromone, as well as the different solutions of other processors. Thus, the pheromone update is done in a central manner. In a decentralized approach, each processor has to calculate the pheromone update itself using the information received from other processors.

PAntOR consists of a standard ACO parallelization (large-grained parallelization) with a decentralized approach.

To understand how PAntOR works, it is necessary to employ three concepts:

(a) Process: program running. The processes are managed by the operating system.
(b) Thread: the basic unit of execution. Any program that executes at least has a thread.
(c) Portable Operating System Interface (POSIX) thread: Standard based on the threads Application Programming Interface (API) for C/C++.

We use POSIX [24,25] thread because it allows a new concurrent process flow to expand. This is the most efficient multi-core system, where the flow of processes can be scheduled to run on another processor, thus gaining speed through parallel or distributed processing. Programming with threads carries less overhead than expanding a new process, because the system does not initialize a new environment and virtual memory space for that process.

Parallel programming technologies, such as Message Passing Interface (MPI) and Parallel Virtual Machine (PVM), are used in a distributed computing environment, while the threads are limited to a single computer system. All threads within a process share the same address space. For the implementation of this routing algorithm to be faster, we use the POSIX thread library.

This parallel technique involves launching a thread for each neighbor that initiates any of the following processes: route setup, local route repair and link failure notification.

As its name suggests, PAntOR-MI [26] is a variant of PAntOR designed for devices that contain more than one interface, i.e., for small and portable devices with more than one antenna or wireless network interface (PocketPC, mobile phones of last generation, and so on).

PAntOR-MI parallelizes the sending ants broadcast through the interfaces using threads. Due to the difficulty of finding specialized hardware, PAntOR-MI only has been applied to the route setup process using two interfaces.

Table 5 shows the main features of the AntOR protocols following a sequential and parallel approach.

## 5. Results

The comparison has been done using the most representative sequential protocols: AntOR-DLR, AntOR-RDLR, AntOR-UDLR, AntOR-v2 and HACOR.

In Appendix A, the simulation and performance metrics’ characteristics are explained.

Table 6 shows the characteristics of the scenario that has been simulated.

Then, the performance metrics are analyzed.

We have used the same seed for each algorithm run with the same randomness pattern. Therefore, the starting and ending time of the algorithm, the RNG and the initial position are the same for all algorithms. Each algorithm has different behaviors, and we can see the differences among algorithms. In the tests, in the same algorithm, each run of five had a similar value of the metric, so that the media were pretty similar.

### 5.1. Average End-To-End Delay

**Figure 4:**
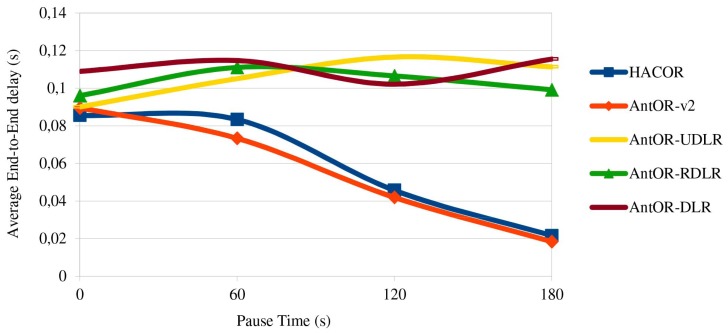
Average end-to-end delay.

Figure 4 shows the average end-to-end delay. The best values are in HACOR and AntOR-v2 by the good management of obsolete routes. This management purges routes, in order for there to be no conflicts, and thus, the data packets can be sent to destinations, better reducing the delay. Furthermore, the delay is improved with the packet buffering control, since the overlapping of messages sent disappears, because the routes are created before, which makes it more efficient. It is observed that for more static scenarios (higher pause), AntOR-RDLR gets a small improvement in delay than AntOR-UDLR, because it allows proactive ants to go through disjoint routes, up to a certain number of attempts. Instead, the curve of AntOR-DLR presents a more irregular behavior, and it has a higher delay in scenarios that are more dynamic.

### 5.2. Jitter

**Figure 5:**
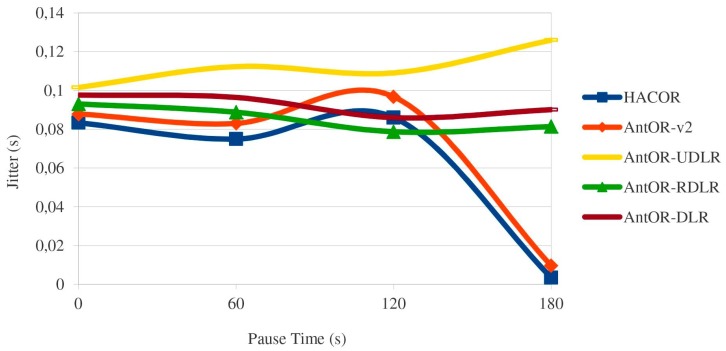
Jitter.

Figure 5 shows the jitter. As can be seen, HACOR and AntOR-v2 are better than their predecessors AntOR-UDLR, AntOR-RDLR and AntOR-DLR. Generally speaking, it is better due to the proactive processes. In HACOR, it is the S-ACO mechanism, and in AntOR-v2, it is sent to neighbor with a higher regular pheromone at the time of calculating the alternatives in proactive ant sent. The AntOR-UDLR behavior makes a certain delay for the reception time of consecutive packets since the nodes have to verify that the channel is free from having to send unicast messages, as shown in figure. In AntOR-DLR, the jitter is slightly higher than AntOR-RDLR, because we do not allow proactive ants to go through disjoint paths. Nevertheless, the two curves have similar behavior.

### 5.3. Overhead in the Number of Bytes

**Figure 6:**
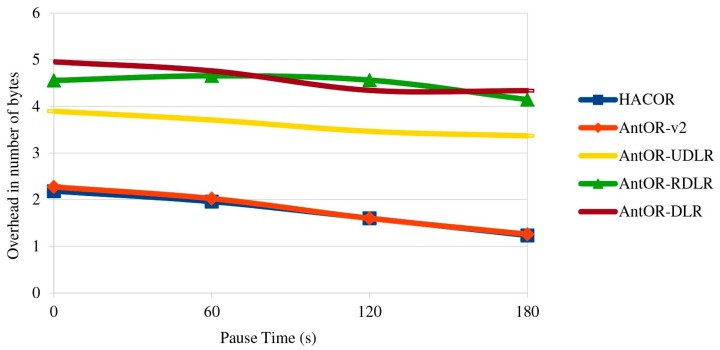
Overhead in the number of bytes.

Figure 6 shows the overhead in number of bytes. HACOR and AntOR-v2 do not use pheromone diffusion because they need the virtual pheromone. This fact makes their respective overload in bytes to be more reduced. In AntOR-UDLR, the sending of unicast agents causes link failures to be neutralized in a more efficient manner, reducing the overhead caused by AntOR-DLR and AntOR-RDLR.

### 5.4. Overhead in the Number of Packets

**Figure 7:**
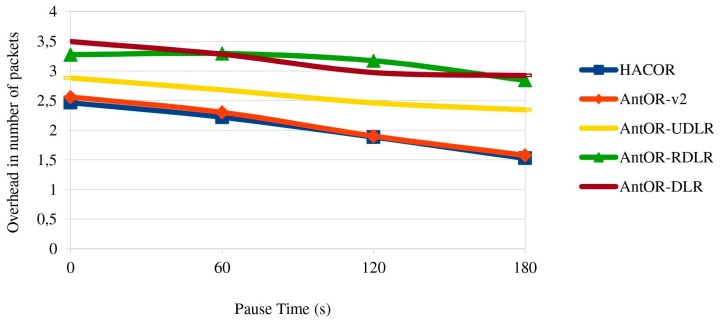
Overhead in the number of packets.

Figure 7 shows the overhead in the number of packets. The results are similar to those of Figure 6. We also see that the overhead is lower in HACOR and AntOR-v2 by the route exploration mechanisms.

### 5.5. Delivered Data Packet Ratio

**Figure 8:**
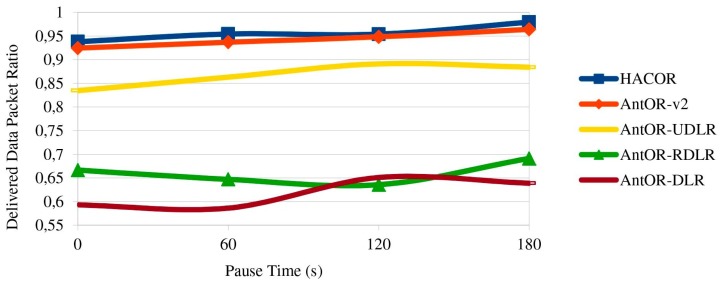
Delivered data packet ratio.

Figure 8 shows the delivered data packet ratio. HACOR and AntOR-v2 get the higher values, due to control packet buffering and route obsolete management. You can also see how in AntOR-UDLR, the ratio is lower than the previous two, but better than AntOR-RDLR and AntOR-DLR, because link failure notification messages sent in broadcast mode are substituted by simple unicast messages sent to the precursor of a valid path since the failure neutralization process is more efficient with unicast messages in AntOR-UDLR. With higher pause times, a better ratio is obtained in all protocols. AntOR-DLR has a worse ratio than other protocols because it is not optimized. We can see that the ratio in AntOR-DLR is lower than AntOR-RDLR in both dynamic scenarios (low pause time) as for static scenarios (high pause time).

### 5.6. Throughput

**Figure 9:**
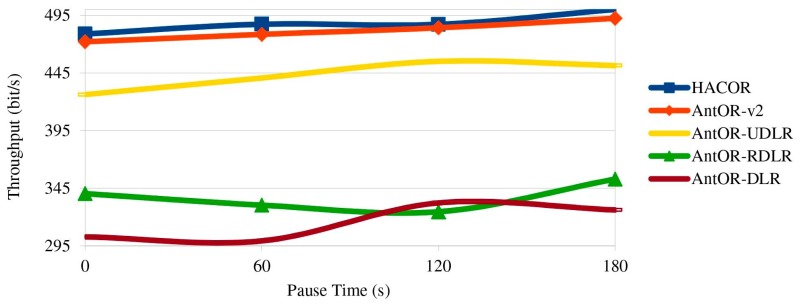
Throughput.

Figure 9 shows the throughput. We can see that it is similar to Figure 8, but using a different scale.

**Figure 10:**
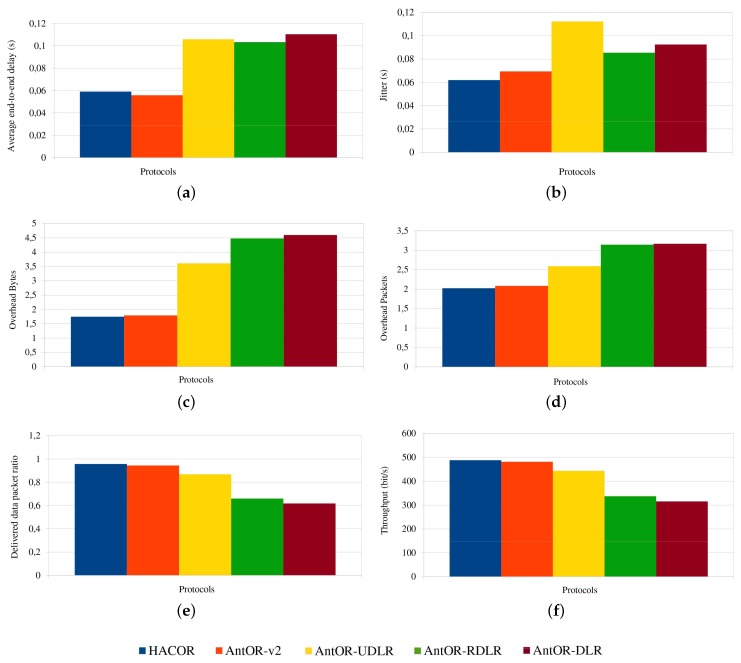
Comparative diagram of the analyzed protocols. (**a**) Average delay; (**b**) average jitter; (**c**) average overhead bytes; (**d**) average overhead packets; (**e**) average ratio; (**f**) average throughput.

Then, in Figure 10, a comparative diagram of the analyzed protocols according to average of the performance metrics is shown. These results are given in a specific scenario, but may vary based on other factors: the number of nodes or node speed.

Table 7 shows a summary scheme with the results of the simulations.

These simulations were performed in a specific scenario and designed considering a sequential approach. HACOR and AntOR-v2 offer the best performance in the delivered data packet ratio by control packet buffering and route obsolete management. Further, HACOR uses data packet buffering to try to allow these packets to reach their destinations without increasing the average end-to-end delay. Furthermore in this protocol, a new link fault management is used to achieve greater fault tolerance, because it repairs routes before and more robustly. Furthermore, the use of S-ACO in the route exploration mechanism gets a good estimate in choosing intermediate neighbor nodes for forwarding proactive agents.

Summarizing: We can check how HACOR and AntOR-v2 have similar behaviors because they share common techniques, such as control packet buffering, route obsolete management and the new route exploration mechanism without using the virtual pheromone. AntOR-DLR and AntOR-RDLR have worse results because they are less evolved protocols because they include few optimization techniques. Finally, AntOR-UDLR has some intermediate results and a high jitter due to the use of link failure notification messages sent in unicast mode.

## 6. Discussion

The main lines of research resulting from this work are:

- The design of new parallelization techniques: It would be interesting to get more efficient parallel implementations.
- Secure extension of the specified protocol: All ACO routing protocols for mobile ad hoc networks are designed without considering the malicious behavior of some nodes, which can be exploited to violate the network security.
- Application of the ACO routing protocols developed for mobile ad hoc networks in other fields: An area of immediate application would be sensor networks given the similarity between both types of networks. Another field of application could be robotics, where frequently, the use of simple local interactions is analyzed to solve complex tasks, such as, for example, navigation in indoor environments.

## 7. Conclusions

This work has addressed a fundamental aspect of so-called mobile ad hoc networks, the routing problem.

First of all, we have specified a new ACO routing protocol for mobile ad hoc networks called AntOR. Like its predecessor AntHocNet, AntOR is hybrid in the sense that it contains both reactive and proactive routing elements. In particular, it combines a reactive process of route setup with a proactive process of the maintenance and exploration of new routes. Routing information is stored in pheromone tables that are similar to those utilized by other ACO routing algorithms. The forwarding of data and control packets is performed in a stochastic manner with the use of these tables. Link failures are treated with specific reactive mechanisms, such as the local route repair and the use of warning messages. The key aspects of the AntOR protocol are the use of disjoint node and disjoint link routes, the separation between the regular pheromone and the virtual pheromone in the diffusion process and the exploration of new routes, which takes into account the number of hops in the best routes.

Then, we have specified a family of ACO routing protocols for mobile ad hoc networks; all of them derived from the AntOR protocol, presenting two variants: the disjoint link version (AntOR-DLR) and the disjoint node version (AntOR-DNR). The disjoint link version has originated a set of sequential protocols: AntOR-RDLR, AntOR-UDLR, AntOR-v2 and HACOR. All of these protocols are successive refinements from the original protocol. The disjoint node version has resulted in a set of parallel protocols: PAntOR and PAntOR-MI.

In AntOR-DNR, the routes do not share nodes and in AntOR-DLR do not share links. By the disjoint property, a failure in one node only affects a path, not the entire network. In addition, load balancing is better (by not repeating routes). The routes’ calculation in AntOR-DLR is easier (less restrictive) than AntOR-DNR since all disjoint nodes also comprise a disjoint link, but not vice versa.

AntOR-RDLR differs from its predecessor (AntOR-DLR) in the pheromone update process and the route discovery mechanism, allowing the proactive forward ants to go by disjoint link routes until a maximum number of attempts has been made. The latter allows the generation of more alternative routes.

The main idea of AntOR-UDLR is to replace link failure notification messages by unicast messages that are sent to the predecessor of the node that reports about the link failure until reaching the source of the data session, since in AntOR-DLR, this is sent in broadcast mode. The use of unicast messages causes losing fewer messages, because before transmitting, it is checked if the medium is available through which you want to send; the fact is that it does not happen when it is sent in broadcast mode. This new protocol aims to reduce network traffic, preventing the transmission of information from unnecessary nodes that do not need to process it.

AntOR-v2 and HACOR are the two more evolved variants, providing new optimization techniques such as the storage of control packets and outdated routes management, as well as different failures link management and route exploration. The main difference between AntOR-v2 and HACOR is in the route exploration process and consists of the technique used. In AntOR-v2, proactive ants are sent to the one-hop neighbor with the best pheromone value. On the other hand, in HACOR, the proactive process focuses on the algorithmic implementation S-ACO, which constitutes the starting point of the functioning of the ACO algorithms. HACOR also presents new techniques of link failure neutralization.

PAntOR is a large-grained parallelization version of AntOR making use of multiprocessor programming architectures based on a shared memory system through the standardization Posixthread, which allows it to execute tasks in parallel using threads, this parallelization being applicable in the route setup phase, local route repair process and link failure notification. PAntOR-MI is a multi-interface variant, which parallelizes the sending of broadcast messages by an interface through threads.

Finally, various simulations have been performed in NS-3 in order to validate the earlier proposals. The simulation results show that: (i) the AntOR-DLR protocol presented better throughput and a better delivered data packet ratio than its predecessor AntHocNet, as well as a small increase of average end-to- end delay and overhead, while these two latest metrics tend to equalize in dense networks; (ii) the AntOR-DLR protocol improves AntOR-DNR; (iii) the AntOR-RDLR protocol improves its predecessor AntOR-DLR; (iv) the AntOR-UDLR protocol also improves its predecessor AntOR-DLR in all metrics with the exception of the overload, in which case, both protocols presented similar values; (v) the AntOR-v2 protocol improves AODV in all of the analyzed metrics, with the exception of the overload, which is slightly superior; this difference becomes undetectable in dense networks; (vi) HACOR protocol improves AODV and OLSR in the throughput and the delivered data packet ratio, showing intermediate values for these two protocols in the other considered metrics; (vii) the PAntOR protocol improves AntOR-DNR; (viii) the PAntOR-MI protocol improves its predecessor in very dynamic environments; and (ix) all specified protocols behave stably in all of the simulations carried out. The previous results allow us to conclude that the family of specified sequential protocols (of which HACOR is its greatest exponent) improves the scalability of its predecessor AntHocNet, a protocol that is also better for most environments than the standards of reactive routing (AODV) and proactive routing (OLSR), and the considered parallel approaches for this type of ACO routing algorithms can further enhance the benefits of this family.

## Figures and Tables

**Table 1:** Scheme of the ACO protocols.

Protocol Property	Type of Routing	Type Routes	QoS	Disjoint	Observations
Adaptive-SDR	Proactive	-	NO	NO	High overhead.
					Resource use.
MABR	Proactive	-	NO	NO	Pheromone evaporation.
					Theory Model.
PERA	Proactive	-	NO	NO	High overhead.
AntNet-RSLR	Proactive	-	NO	NO	High overhead.
ARA	Reactive	-	NO	NO	No multimedia data.
ADRA	Reactive	-	YES	NO	Pheromone evaporation.
					Better performance than DSR.
PACONET	Reactive	-	NO	NO	Probabilistic Algorithm.
Ant-AODV	Hybrid	-	NO	NO	Less latency and end-to-end delay.
AntHocNet	Hybrid	Multipath	NO	NO	Adaptive.
					Equal or better performance than AODV.
AntHocNet	Hybrid	Multipath	NO	NO	Less overhead.
(Ducatelle thesis)					
HOPNET	Hybrid	-	NO	NO	With few nodes more delay than other hybrids.
AntOR	Hybrid	Multi-path	NO	YES	Pheromone separation.
					Distance metric.

**Table 2:** Routing table (AntOR).

Entries	Destination	Next Hop	Regular Pheromone Value (τ)	Virtual Pheromone Value (ω)	Average Number of Hops (h)
$Entry_{1}$	$Destination_{1}$	Next $Hop_{1}$	$\tau_{1}$	$\omega_{1}$	$h_{1}$
$Entry_{2}$	$Destination_{2}$	Next $Hop_{2}$	$\tau_{2}$	$\omega_{2}$	$h_{2}$
⋯	⋯	⋯	⋯	⋯	⋯
$Entry_{i}$	$Destination_{i}$	Next $Hop_{i}$	$\tau_{i}$	$\omega_{i}$	$h_{i}$
⋯	⋯	⋯	⋯	⋯	⋯

**Table 3:** Neighbor table (AntOR).

Local node	Id $Neigh_{1}$	Id $Neigh_{2}$	⋯	Id $Neigh_{k}$	⋯	Id $Neigh_{N}$
	Time $Neigh_{1}$	Time $Neigh_{2}$		Time $Neigh_{k}$		Time $Neigh_{N}$

**Table 4:** Routing table (AntOR-DLR).

Entries	Destination	Next Hop	Regular Pheromone Value (τ)	Virtual Pheromone Value (ω)	Average Number of Hops (h)	Disjoint Session (s)
$Entry_{1}$	$Destination_{1}$	Next $Hop_{1}$	$\tau_{1}$	$\omega_{1}$	$h_{1}$	$s_{1}$
$Entry_{2}$	$Destination_{2}$	Next $Hop_{2}$	$\tau_{2}$	$\omega_{2}$	$h_{2}$	$s_{2}$
⋯	⋯	⋯	⋯	⋯	⋯	⋯
$Entry_{i}$	$Destination_{i}$	Next $Hop_{i}$	$\tau_{i}$	$\omega_{i}$	$h_{i}$	$s_{i}$
⋯	⋯	⋯	⋯	⋯	⋯	⋯

**Table 5:** Characteristics of protocols based on AntOR.

Protocol/Characteristic	Approximation	Route Obsolete Management	Control Packet Buffer	Disjoint	Other Characteristics
AntOR	-	NO	NO	Node/link	Pheromone separation.
					Distance metric.
AntOR-DLR	-	NO	NO	link	-
AntOR-DNR	-	NO	NO	Node	-
AntOR-RDLR	Sequential	NO	NO	Link	Flexibility sent of agents through disjoint routes.
AntOR-UDLR	Sequential	NO	NO	Link	Link failure unicast Management.
AntOR-v2	Sequential	YES	YES	Link	No virtual pheromone.
					New route exploration.
HACOR	Sequential	YES	YES	Link	No virtual pheromone.
					Route exploration based on S-ACO.
					New neutralization process.
P-AntOR	Parallel	NO	NO	Node	Based on thread.
PAntOR-MI	Parallel	NO	NO	Node	Based on thread.
					Multi-interface.

**Table 6:** Scenario characteristics.

Parameter	Value
Number of nodes	100 nodes
Node distribution	Random
Dimensions of area	2000 m × 1500 m
Time simulation	180 s
Physical layer	IEEE 802.11
Transmission range	300 m
Number of runs	5
Traffic generator	Constant bit rate (CBR)
Beginning of time CBR client	uniform distribution [0–60] s
Ending of time CBR client	180 s
Beginning of time CBR server	0 s
Ending of time CBR server	180 s
Number of data sessions	10
Data rate	512 bits/s (1 packets of 64 bytes per second)
Mobility pattern	Random waypoint (RWP)
Node speed	[0–5] m/s

**Table 7:** Results of the protocols.

	Protocols	DLR	RDLR	UDLR	AntOR-v2	HACOR
Results						
**Delay**		HIGH	HIGH	HIGH	LOW	LOW
**Jitter**		MEDIUM	MEDIUM	HIGH	LOW	LOW
**Overhead in bytes**		HIGH	HIGH	MEDIUM	LOW	LOW
**Overhead in packets**		HIGH	HIGH	MEDIUM	LOW	LOW
**Ratio**		LOW	LOW	MEDIUM	HIGH	HIGH
**Throughput**		LOW	LOW	MEDIUM	HIGH	HIGH

## Appendix A

Network Simulator 3 (NS-3) [27]: Similar to its predecessor NS-2 [28], it is a discrete event simulator and is based on C++ for the implementation of models of the simulation. However, NS-3 does not utilize command scripts oTcl anymore to control the simulation, avoiding the problems presented by the combination of C++ and oTcl in NS-2. Simulation scenarios in NS-3 can be implemented in pure C++, and also, it could use Python.

### Performance Metrics

To evaluate these routing protocols, we have divided the metrics into effectiveness and efficiency. The first measures the performance: throughput, delivered data packet ratio, average end-to-end delay and jitter. The second refers to generated overhead: overhead in packets and overhead in bytes.

- Throughput: the volume of work or information flowing through a system. It is calculated by dividing the total number of bits delivered to the destination by the packet delivery time.
- Delivered data packet ratio: the relationship between the number of packets delivered successfully and the number of packets sent.
- Average end-to-end delay: the average time of the transmission of a data packet in the network from the source to destination.
- Jitter: the measurement of the variation of the time of arrival of consecutive data packets.
- Overhead in the number of packets: the relationship between the number of transmitted control packets and the number of successfully delivered data packets.
- Overhead in the number of bytes: the relationship between the total number of bytes sent and the number of bytes of the correctly delivered data packets.
